# Supplementary material for: Analysis of Volatile Aroma Components and Regulatory Genes in Different Kinds and Development Stages of Pepper Fruits Based on Non-Targeted Metabolome Combined with Transcriptome
Source: Int J Mol Sci. 2023 Apr 26;24(9):7901. doi: 10.3390/ijms24097901 (PMC10178352; doi:10.3390/ijms24097901)
Supplement: Supplementary file 1 [file ijms-24-07901-s001.zip › Supplementary Figures.pdf]

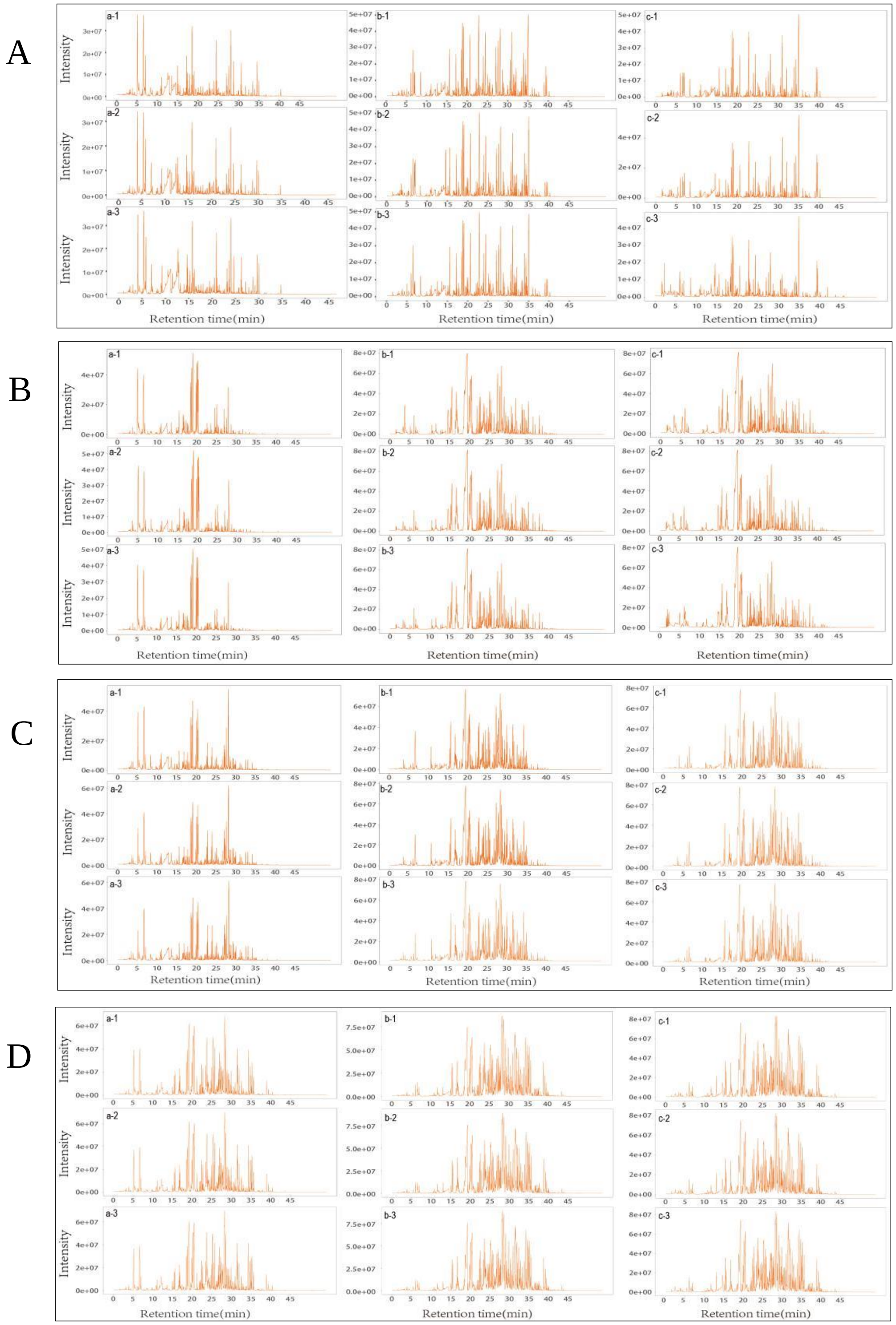

Figure S1. (A): Gas chromatogram of CTJ volatile compounds; (B): Gas chromatogram of GJ volatile compounds; (C): Gas chromatogram of HDL 1 volatile compounds; (D): Gas chromatogram of HDL 2 volatile compounds

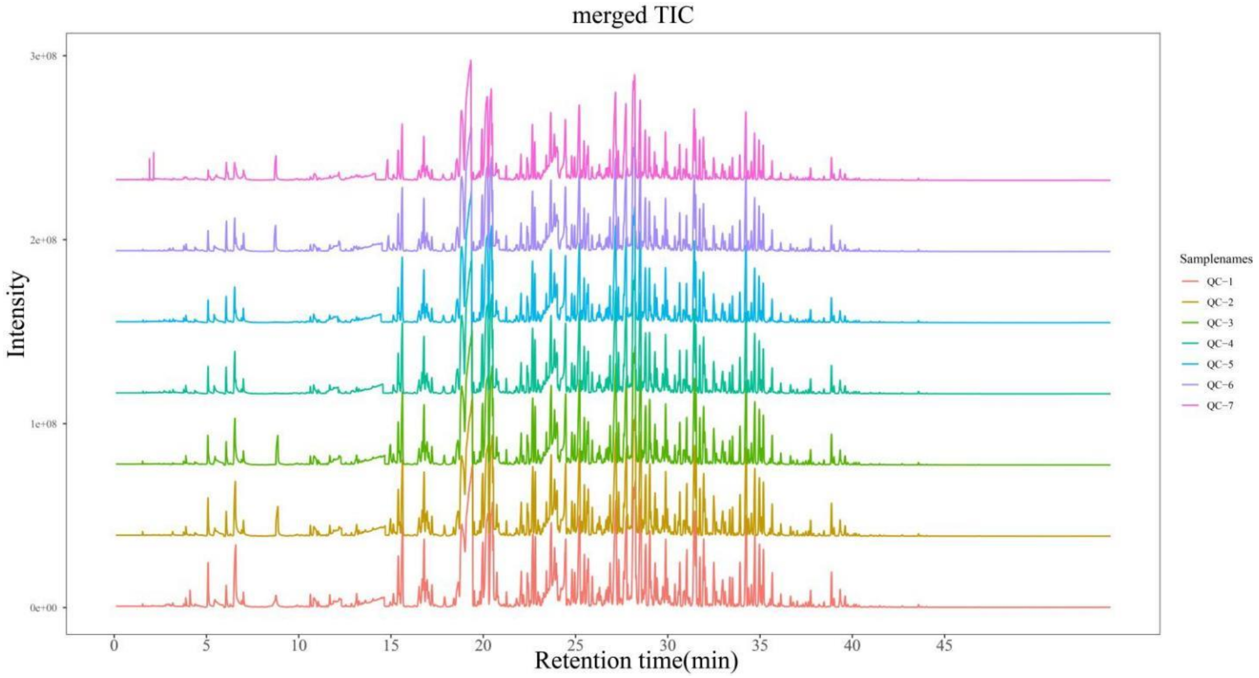

Figure S2. QC sample TIC superposition diagram

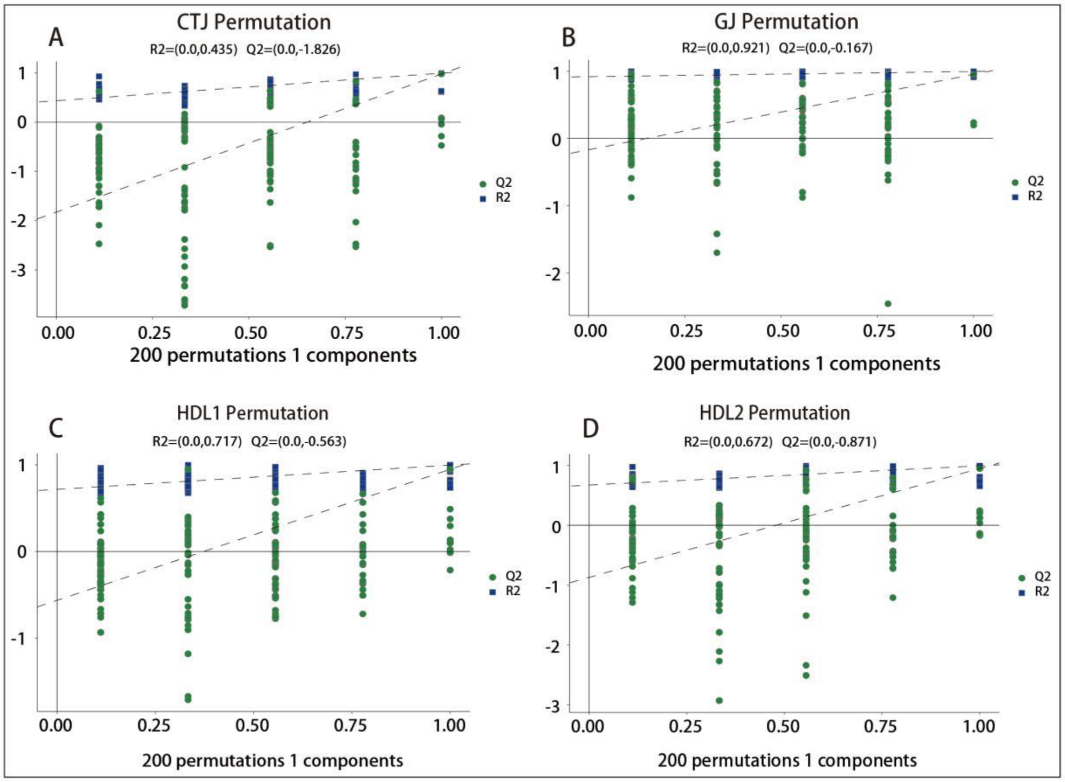

Figure S3. Results of model validation built by PLS-DA under different comparisons during the growth of four pepper varieties. (A): CTJ permutation test plot; (B): GJ permutation test plot; (C): HDL1 permutation test plot; (D): HDL2 permutation test plot.

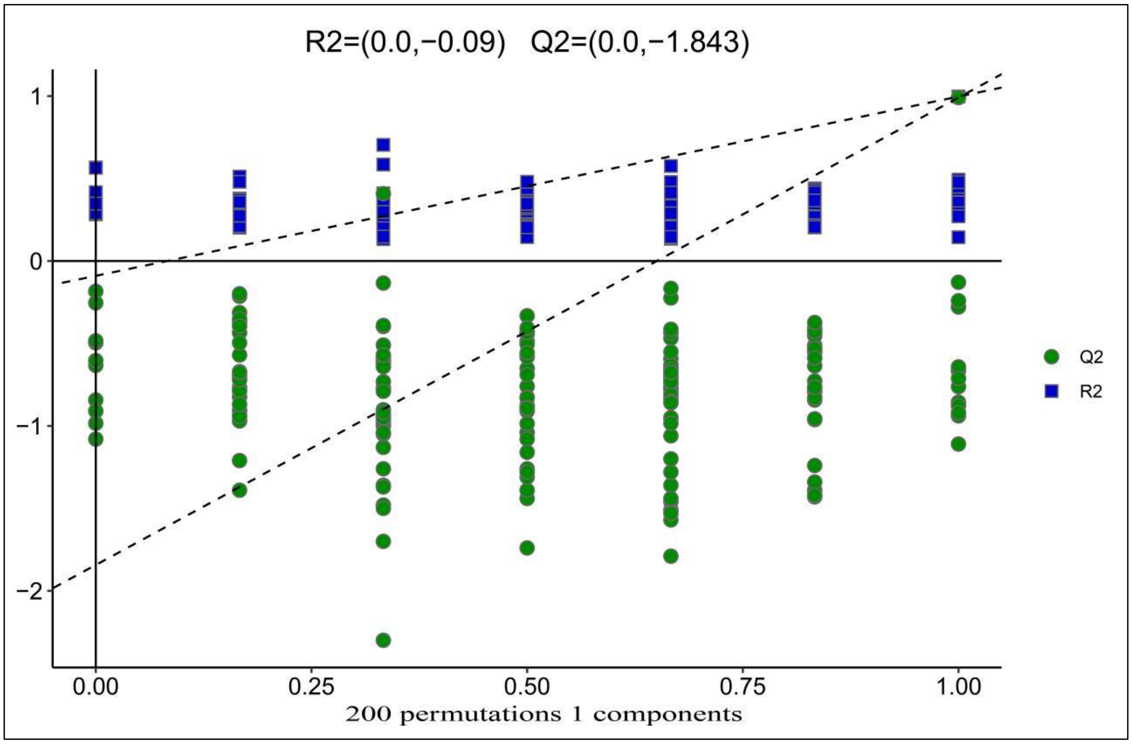

Figure S4. Results of model validation established by PLS-DA during the ripening stage of four pepper varieties.

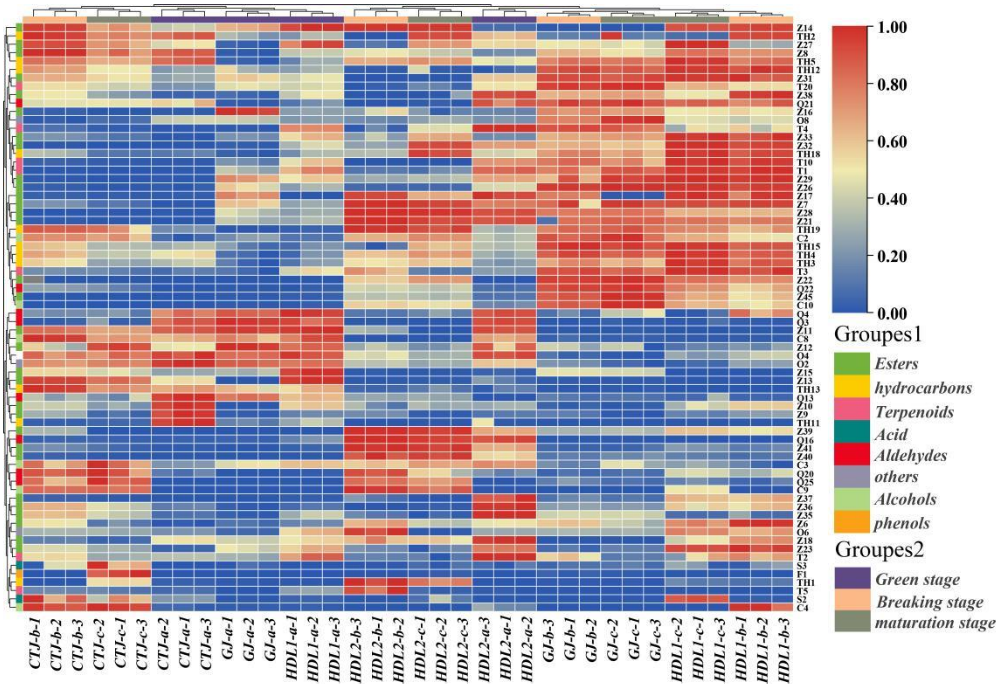

Figure S5. Heatmap of the 70 differential aroma compounds (VIP> 1, $p < 0.05$ and $|\log2FC| \geq 1$) in the different comparison groups(μg/kg).

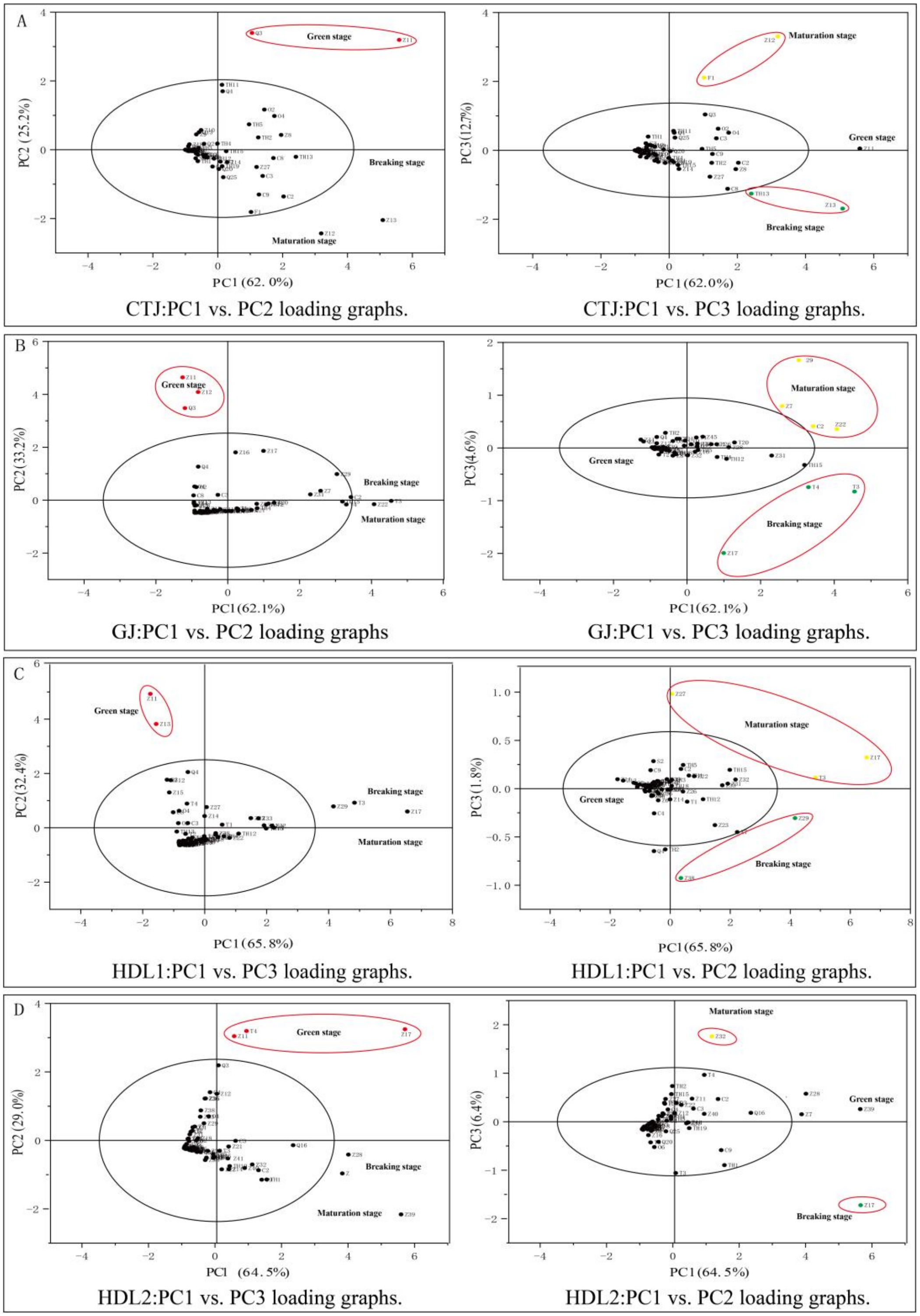

Figure S6. Characteristic marker aroma compounds in pepper fruits at different growth periods. (A) marker aroma compounds at the different growth stages of the CTJ; (B) marker aroma compounds at the different growth stages of the GJ; (C) marker aroma compounds at the different growth stages of the HDL1; (D) marker aroma compounds at the different growth stages of the HDL2.

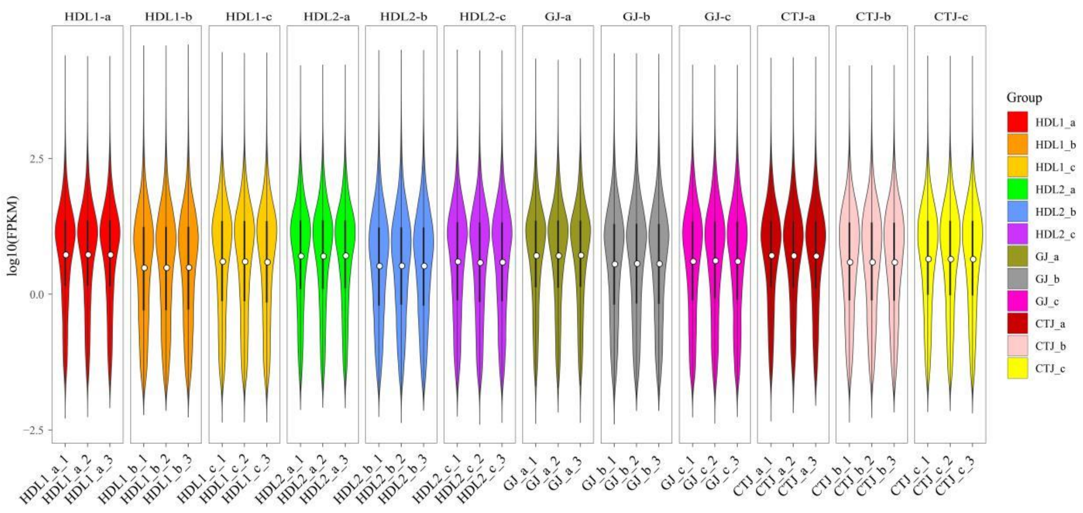

Figure S7. Violin plots of the FPKM values for the genes in each sample.

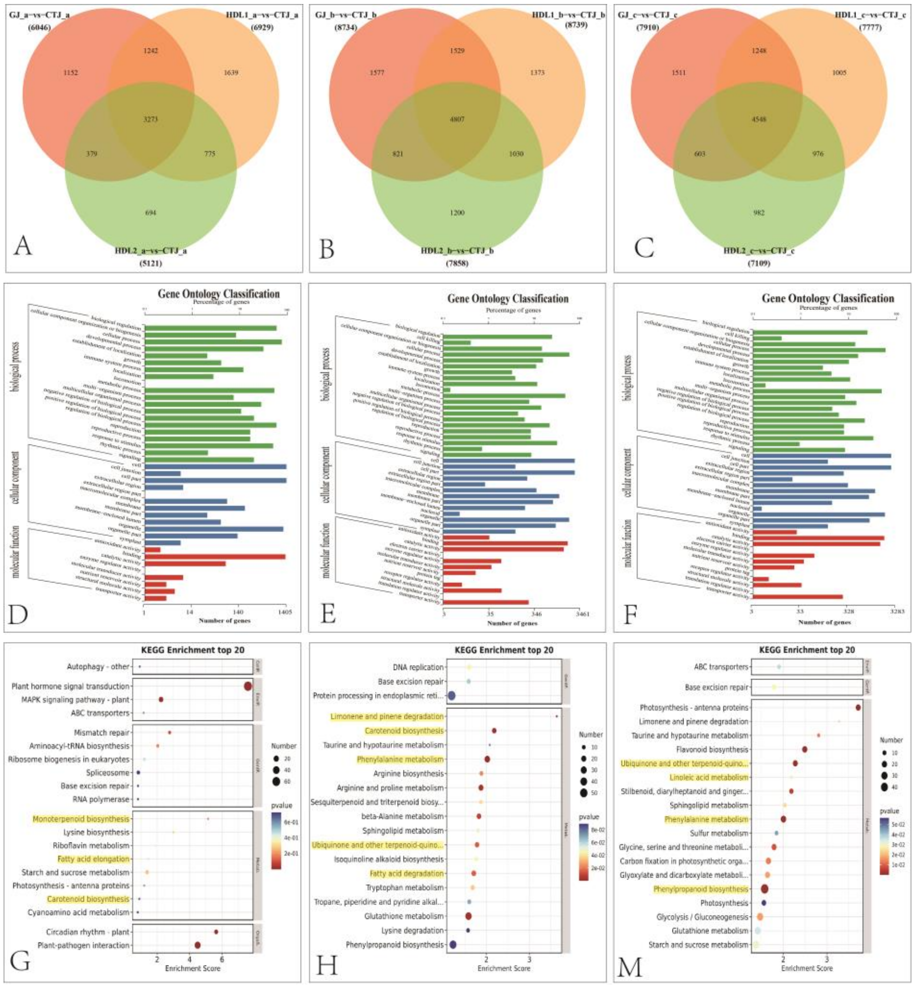

Figure S8. Functional enrichment analysis of differentially expressed genes (DEGs) between variety comparisons; (A-C) Venn diagram of the DEGs in different varieties of peppers compared during the same; (D-F) Gene ontology

(GO)classifications of overlapping DEGs in a, b, and c; (G-M) Kyoto Encyclopedia of Genes and Genomes (KEGG) pathway enrichment of DEGsin a, b and c.

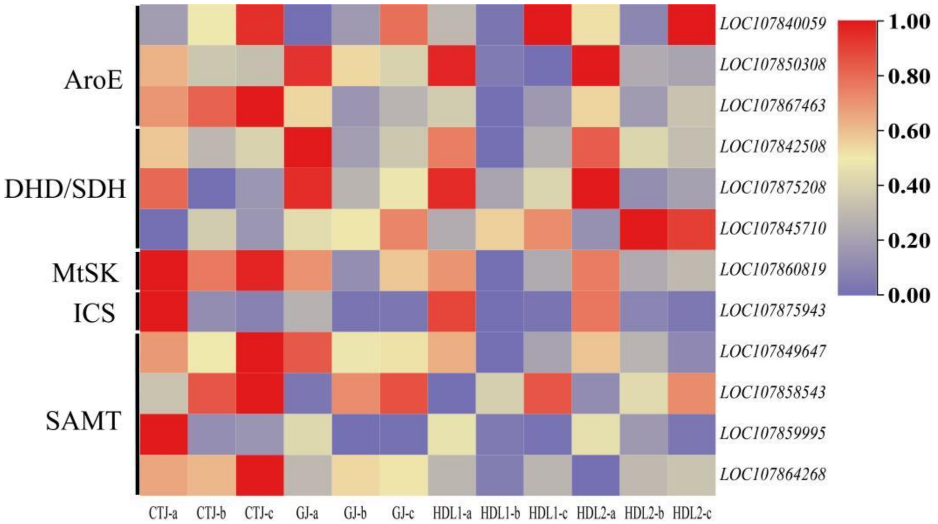

Figure S9. Heatmap of key differential gene expression patterns associated with methyl salicylate synthesis during different growth periods of the four pepper varieties. AroE: phospho-2-dehydro-3-Deoxyheptonate aldolase; DHD/SDH:bifunctional 3-dehydroquinate dehydratase/shikimate dehydrogenase; MtSK: shikimate kinase; ICS: isochorismate synthase;SAMT:salicylate carboxymethyl transferase

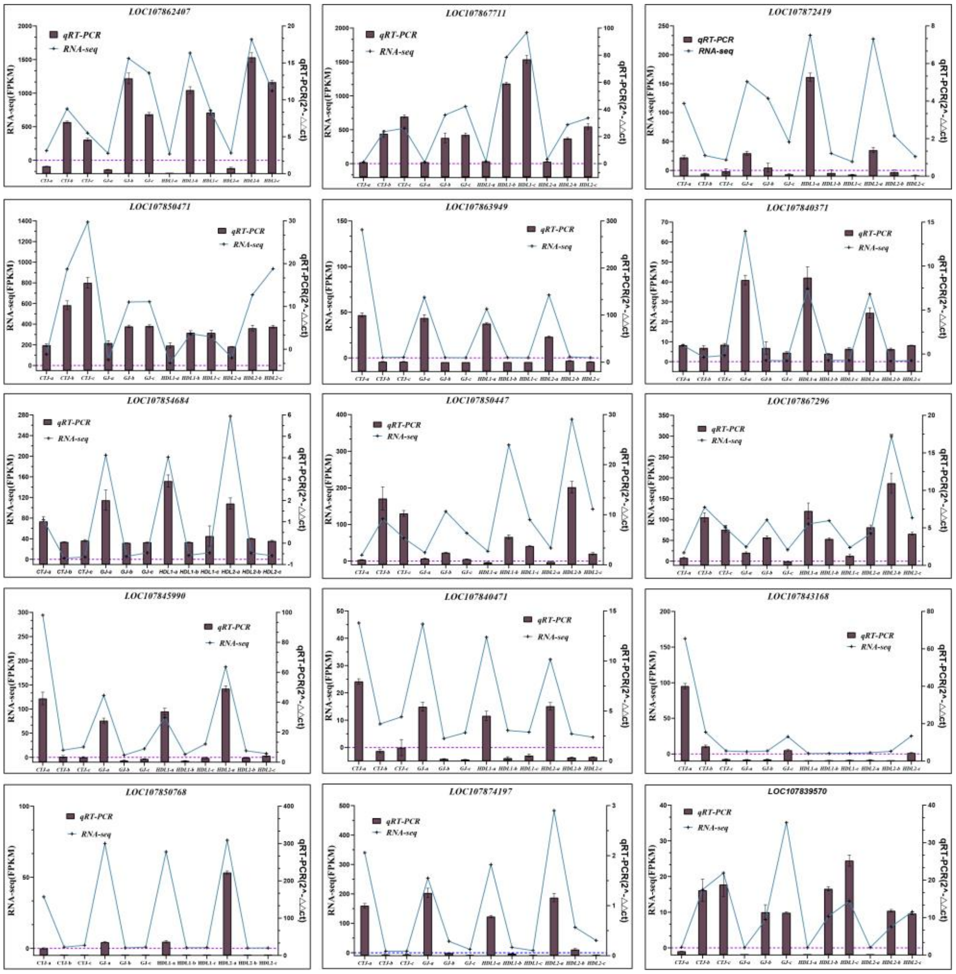

Figure S10. qRT-PCR validated candidate genes associated with the synthesis of volatile aroma compounds Error bars show±SE from three biological replicates.
